# The Impact of En-bloc Transurethral Resection of Bladder Tumour on Clinical, Pathological and Oncological Outcomes: A Cohort Study

**DOI:** 10.7759/cureus.42523

**Published:** 2023-07-26

**Authors:** Deerush Kannan, Praveen G Sekaran, Sindhu Sankaran, Pratik Taur, Sanjay Prakash J, Rajesh Paul, Mathisekaran Thangarasu, Nitesh Jain

**Affiliations:** 1 Urology, Apollo Hospitals, Chennai, IND; 2 General Surgery, Saveetha Medical College and Hospital, Chennai, IND; 3 Urology, Addenbrooke's Hospital, Cambridge University Hospitals National Health Services (NHS) Foundation Trust, Cambridge, GBR; 4 Urology, Asian Institute of Nephrology and Urology, Chennai, IND

**Keywords:** bladder cancer, outcomes, turbt, eturbt, en-bloc transurethral resection of bladder tissue

## Abstract

Background

En-bloc transurethral resection of bladder tissue (ETURBT) has recently been proposed as a good alternative technique to trans-urethral resection of bladder tissue (TURBT) in terms of outcomes for bladder carcinoma. This study aims to assess the effectiveness of the technique in terms of clinical, pathological and oncological outcomes.

Methodology

In this prospective study, data was collected from patients who underwent ETURBT for bladder space-occupying lesions between June 2021 and June 2022. Demographic characteristics, tumour characteristics, and postoperative outcomes were recorded.

Results

A total of 52 patients were studied with the majority being male and a mean age of 50.87 years. Smoking was recorded in 22 (38.5%) patients and 8 (15.4%) were on antiplatelet therapy. The majority fell in the American Society of Anesthesiology (ASA) class I (59.6%). Most of the tumours were solitary (90.4%), primary (82.8%), papillary architecture (73.1%), and between 1-3 cm in size. The lateral wall was the most common position, and detrusor muscle was seen in 98.1% of the specimens. T1 stage (57.7%) and low grade (67.3%) were the common characteristics noted. 76.9% of the ETURBT was conducted using monopolar cautery. Recurrence was noted in 3 (5.8%) and bladder perforation in 1 patient (1.9%). Cautery artifact was seen in six patients (11.5%) and obturator jerk in nine patients (17.3%).

Conclusion

Our study suggests that ETURBT is a technique with a good success rate for bladder tumours less than 3 cm in size. The benefits include high chances of detrusor sampling while minimising crush artefacts and cautery damage. Specimen retrieval was challenging when the bladder tumour was solid and over 2 cm.

## Introduction

Urinary bladder carcinoma is the fourth most common malignancy in men and the eighth most common malignancy in women in the Western world. Around 5% to 10% of all malignancies in men in Europe and the United States are bladder cancers [1]. Bladder cancer is the ninth most common cancer accounting for 3.9% of all cancer cases as per the Indian Cancer Registry data. Data on the exact incidence is scarce [2].

The primary management in all bladder space-occupying lesions includes a complete transurethral resection of the bladder tumour (TURBT) and a histopathological examination by a pathologist depending on which further treatment is planned. While TURBT is the gold standard, this mode of resection violates the typical oncological principles, due to the implantation of scattered and exfoliated tumour cells from the “piece-meal” resection. This has been associated with increased recurrence [3]. Other limitations include unclear resection margins and the inability to ensure the inclusion of detrusor muscle in the final histopathology sample. These factors decrease the accuracy of staging and oncological outcomes of non-muscle invasive bladder cancer (NMIBC) [4]. To overcome these difficulties, en-bloc transurethral resection of bladder tumour (ETURBT) has been proposed.

First described in 1997, ETURBT involves removing the tumour in a “one-piece” fashion. This involves a circular incision either with blade or energy devices around the tumour followed by removal in toto with the underlying detrusor muscle [5]. The advantages include maintenance of the 3D architecture of the tumour enabling accurate staging of bladder cancer and proper assessment of the margins of the tumour [6]. Remaining in the same surgical plane decreases complications. Avoiding tumour fragmentation decreases tumour spillage and improves oncological outcomes [7]. When a good assessment of the depth of invasion is possible, it can avoid re-look TURBT even in T1 tumours or high-grade tumours [5,8]. This especially helps extend the scope of this novel procedure in pathologies that were previously not treated by TURBT definitively which includes conditions like muscle-invasive bladder cancer (MIBC) and carcinoma-in-situ (CIS).

Very few studies have addressed the outcomes of ETURBT, especially in our population. In this study, we are performing a prospective analysis to assess the effectiveness of ETURBT in bladder tumours in terms of completeness of resection based on post-op histopathology and incidence of early and late complications.

## Materials and methods

This was a single-centre prospective study conducted in Apollo Hospitals, Chennai, India, between June 2021 and June 2022, involving the population presenting to the urology outpatient department. This study was conducted according to the ethical guidelines of the Declaration of Helsinki and its amendments. The study has been approved by our Institutional Ethics Committee (approval number: AMH-DNB-050/06-21) on 7th June 2021. All patients participating in this study provided informed consent. The authors confirm the availability of, and access to, all original data reported in this study.

The study included patients more than 18 years of age who underwent ETURBT for a new or recurrent space-occupying lesion in the bladder based on imaging or surveillance-detected recurrent NMIBC; single and multiple tumours seen on cystoscopy with a size of single tumour less than 3 cm. Exclusion criteria included tumours greater than 3 cm, tumours close to the ureteric orifice, restaging TURBTs for high-grade bladder cancer, patients not willing to participate, benign findings on histopathology, advanced disease on preop imaging and patients who required other simultaneous procedures like transurethral resection of the prostate or ureteroscopy.

The procedure was conducted in the operating room under general or spinal anaesthesia. A detailed cystoscopic evaluation was carried out in all cases. The bladder lesions were meticulously assessed and all data including site, size, multiplicity, relation to the ureteric orifices, and the appearance of the rest of the bladder mucosa were recorded. All resections were carried out in standard fashion either by laser or by monopolar cautery [5]. 

A circular incision was made 5-10 mm around the tumour. Blunt dissection was carried out with care taken to include the underlying detrusor muscle. Small tumours were retrieved with biopsy forceps while in certain cases, Ellick’s bladder evacuator was used to retrieve the specimen. All the visible lesions were resected and the tissue was sent for histopathological examination. The biopsy chips were analysed by a dedicated pathologist and reported based on the presence of muscle in the sample, grade, stage and presence of cautery artifact. 

Complications like catheter block secondary to bladder clots needing transfusion and signs of bladder perforation were looked out for in the initial few days after the procedure. The patient was discharged from the hospital when urine was clear after stopping irrigation for three hours. Repeat haemoglobin was performed in all patients 48 hours after the procedure. Foley was removed on postoperative day (POD) two if urine was clear. A follow-up cystoscopy was done on the third month for all patients to look for recurrence. 

Statistical analysis was done using SPSS version 25.0 (IBM Corp., Armonk, NY). All continuous variables were tested for normality using Shapiro-Wilk's test. If they were normally distributed, then they are expressed as mean SD, otherwise median (IQR). Categorical variables are expressed as a percentage. Comparison of categorical variables was done by Chi-square test and continuous variables by paired t-test. Data entry was done through Microsoft Excel. All p values <0.05 was considered statistically significant.

## Results

Fifty-eight patients with a mean age of 50.87 years and a standard deviation of 11.94 years underwent ETURBT during the study period. Patients were included on an accrual basis. The majority were male (61.5%). Six patients were lost to attrition. Twenty-two patients had comorbidities and 20 were smokers. Eight patients were under antiplatelet therapy. The majority of patients fell in the American Society of Anesthesiology (ASA) class I. The demographic data are given in Table 1.

**Table 1:** Demographic data ASA, American Society of Anesthesiology

Demographics	n=52 (%)
Gender (male/female)	32 (61.5%)/20 (38.5%)
Age (years)	
<40	13 (25%)
41-50	12 (23.1%)
51-60	15 (28.8%)
61-70	9 (17.3%)
>70	3 (5.8%)
Comorbidities	22 (42.3%)
Smokers	20 (38.5%)
On anti-platelets	8 (15.4%)
ASA	
I	31 (59.6%)
II	19 (36.5%)
III	2 (3.8%)

Tumour characteristics

Most of the tumours were primary (82.8%). There were up to three tumours in a single patient, with the majority being solitary tumours (90.4%). Papillary tumours were the majority (73.1%) and sizes of all the tumours varied from 1-3 cm. The most common site was noted to be the lateral wall. Of the tumours removed, 98.1% had detrusor muscle in the specimen and 57.7% were T1 staged. Most of the tumours were of low grade (67.3%). The commonly used energy device in our study was monopolar cautery (76.9%). The tumour characteristics are given in Table 2.

**Table 2:** Tumour characteristics ETURBT, En-bloc Transurethral resection of bladder tumour

Tumour characteristics	n=52 (%)
Type of tumours	
Primary	42 (82.8%)
Recurrent	10 (19.2%)
Number of tumours	
1	47 (90.4%)
2	4 (7.7%)
3	1 (1.9%)
Morphology of tumour	
Papillary	38 (73.1%)
Sessile	14 (26.9%)
Size of tumour (cm)	
1-2	32 (55.2%)
2-3	26 (44.8%)
Site of tumour	
Left lateral wall	16 (30.8%)
Right lateral wall	16 (30.8%)
Posterior wall	9 (17.3%)
Dome	6 (11.5%)
Trigone	5 (9.6%)
Detrusor muscle in the specimen	
Yes	51 (98.1%)
No	1 (1.9%)
T staging	
Ta	14 (26.9%)
T1	30 (57.7%)
T2	8 (15.4%)
Grade of tumour	
Low	35 (67.3%)
High	17 (32.7%)
Procedure	
Monopolar ETURBT	42 (76.9%)
Laser ETUBRT	12 (23.1%)

Postoperative outcomes

Cautery artifact was noted in 11.5% of patients, obturator jerk in 17.3%, and bladder perforation in one patient (1.9%). Follow-up cystoscopy was normal in 71.2% of the patients. Most patients needed three bottles of normal saline irrigation to clear urine, and foley catheter removal was done most commonly on postoperative day (POD) two (61.5%). Table 3 details the postoperative outcomes measured.

**Table 3:** Postoperative outcomes POD, Post-operative day

Postoperative outcomes	
Follow up cystoscopy	
Normal	37 (71.2%)
Recurrence	3 (5.8%)
Cautery artifact	6 (11.5%)
Obturator jerk noted	9 (17.3%)
Bladder perforation	1 (1.9%)
Normal saline irrigation (bottles)	
6	3 (5.8%)
5	5 (9.6%)
4	7 (13.5%)
3	19 (36.5%)
2	16 (30.8%)
1	2 (3.8%)
Foley removal (POD)	
1	2 (3.8%)
2	32 (61.5%)
3	16 (30.8%)
5	1 (1.9%)
10	1 (1.9%)

Since there were multiple tumours in the same patient, the total number of tumours in the study here is 58. In the subgroup of patients who had multiple tumours, all the tumours were present in the same area. All obturator jerks were experienced while resecting the lateral wall tumour. The bladder perforation noted was in the patient who had two tumours in the left lateral wall. One patient who had a tumour in the trigone of size 2 cm and resected with monopolar cautery was noted to have clot retention. Foley was removed on POD 10 for the patient who had bladder perforation.

## Discussion

The endoscopic surgical management of NMIBC has been almost exclusively carried out by TURBT over the last 60 years [9]. During this resection process, cancer cells are released which have been shown to re-implant particularly at sites of surgical injury, which are the top contributing factor to recurrence [10-12]. In contrast, ETURBT allows bladder tumour removal in one piece. The main limitation of the studies describing ETURBT is that they describe a per-protocol series, which introduces a selection bias. 

On the other hand, in certain patients (e.g., BCG-refractory NMIBC, MIBC patients), even a conventional TURBT cannot be regarded as a definitive surgery, and the patients may end up requiring additional intervention in the form of radical cystectomy. Given that ETURBT is now a well-established technique we feel that reporting outcomes based on the routine implementation of ETURBT is more informative for urologists considering adopting the procedure. 

In our study, 61.5% were male patients and the remaining 38.5% were females. Other studies have reported a threefold to a fourfold higher risk of bladder cancer in males as compared to females [13,14]. In our cohort, while men didn’t surpass women by a huge margin, they still accounted for an almost two-fold higher risk than females. In other studies, the average age was 60.2 ± 4.4 years old, while in our participants it was 50.8 years which was almost 10 years less and there was no patient less than 29 years in our study [15]. 

Compared to other studies reporting smoking as a risk factor, our cohort had 61.5% smokers while other studies have reported an incidence of 80.7% including former smokers [16]. About 82.8% of our patients presented with painless haematuria compared to 97% in a study by Gupta et al. [15]. However, our cohort included 19.2% of patients who were referred to our department because of an incidentally detected bladder mass during health check screening. Other studies report at least one comorbid condition in 66% of their study sample while our cohort reported considerably less with 42.3% [17]. About 15.4% of our population were on antiplatelet medications for their heart disease.

Our study only included tumours less than 3 cm in size; although some studies estimated that ETURBT is not feasible for almost 30% of tumours due to size, morphology, and/or location, laser ETURBT has been performed for tumours up to 4.5 to 5.5 cm in diameter and in virtually all locations throughout the bladder [18,19].

While our study included tumours in all locations, we experienced difficulty with anterior wall tumours which could not be resected by ETURBT. Resection was attempted with monopolar current and subsequently required conversion to the conventional method in those cases. These were not included in our study. Tumours in the dome were resected both by laser and monopolar TURBT without any conversions into conventional TURBT. In the study, 42 (76.9%) patients underwent monopolar TURBT and 12 (23.1%) patients underwent laser TURBT. The energy source was selected based on the surgeon's preference.

Studies have compared the different energy sources for ETURBT and concluded that no difference was found in staging and diagnosis of bladder cancers, as all energies ensure a high-quality specimen [20]. In the current cohort, 17.3% of patients had an obturator jerk at the time of tumour resection and all the jerks were experienced while resecting the lateral wall tumours. 

In the trial comparing energy sources for ETURBT, a higher rate of jerk was experienced in lateral wall tumours using monopolar or bipolar energies as compared to a laser source [20]. In the current cohort, the numbers are less for a comparative analysis though it is important to note that the only patient who had perforation underwent resection with a monopolar current for multiple lateral wall tumours.

We faced difficulty while retrieving the resected specimen in tumours more than 2 cm. This is in line with the finding by Naselli et al. who observed that solid lesions are more difficult to extract than papillary lesions. A similar difficulty was reported for lesions arising from the bladder neck [21]. An 18-22 Fr three-way bladder catheter was deployed at the end of the procedure, and continuous bladder irrigation was started. Early one-shot instillation of 40 mg mitomycin C was administered according to current guidelines, but not given in case of bladder wall perforation or excessive bleeding. In the current cohort, one patient had clot retention due to haematuria. Patients followed the postoperative care and follow-up protocols of our institution in line with current European Association of Urology NMIBC guidelines [22]. 

While other studies have used the presence of muscularis mucosae to improve the accuracy of T1 substage, our study did not include this [6]. Similar to observations by Truong et al, we have also noticed significant interference of cautery artefact with the staging [23].

ETURBT has been described as a potential solution to increase the detrusor muscle sampling rate. In the current cohort, 98.1% of samples showed the presence of detrusor muscle in the specimen. Similar rates have been reported in other studies [3,24]. ETURBT can avoid cautery damage and crush artefacts to the specimen. Tangential tissue sections and random embedding of the tumour tissue can also be avoided [25].

Whether ETURBT plays a role in reducing the recurrence rate of NMIBC is controversial. In the current cohort, follow-up cystoscopy was normal in 71.2% of patients and 5.8% of patients had a recurrence. The remaining 23% of the patients had to undergo other modalities of treatment based on their pathology. Some studies have shown no difference in recurrence rate [26,27]. In a study by Sureka et al., ETURBT recurrence-free survival (RFS) was 45.1 months compared to 28.5 months in those who underwent TURBT [8].

Limitations of our study include a short follow-up period and the absence of comparison among the various energy devices for ETURBT or with conventional TURBT. Resection margins could have been evaluated in detail as more importance was given to detrusor muscle and cautery artifacts in our study.

There is limited high-quality prospective trial data on the recurrence rates following ETURBT although the results of ETURBT are promising. A peep into this area of outcome assessment would be a great tool to study the long-term outcome. 

## Conclusions

ETURBT can be a good technique with an excellent success rate for carefully selected tumours. Ideally, tumours smaller than 3 cm can undergo this type of resection. High caution is needed for those located in the anterior wall of the bladder. The advantages are a high chance of detrusor sampling and a lower risk of cautery artefacts. Further studies with randomisation are needed to understand long-term oncological outcomes for non-muscle invasive bladder cancer.

## References

[1] Kirkali Z, Chan T, Manoharan M (2005). Bladder cancer: epidemiology, staging and grading, and diagnosis. Urology.

[2] Prakash G, Pal M, Odaiyappan K (2019). Bladder cancer demographics and outcome data from 2013 at a tertiary cancer hospital in India. Indian J Cancer.

[3] Zhang J, Wang L, Mao S (2018). Transurethral en bloc resection with bipolar button electrode for non-muscle invasive bladder cancer. Int Urol Nephrol.

[4] Herrmann TR, Wolters M, Kramer MW (2017). Transurethral en bloc resection of nonmuscle invasive bladder cancer: trend or hype. Curr Opin Urol.

[5] Kramer MW, Rassweiler JJ, Klein J (2015). En bloc resection of urothelium carcinoma of the bladder (EBRUC): a European multicenter study to compare safety, efficacy, and outcome of laser and electrical en bloc transurethral resection of bladder tumor. World J Urol.

[6] Liang H, Yang T, Wu K, He D, Fan J (2019). En bloc resection improves the identification of muscularis mucosae in non-muscle invasive bladder cancer. World J Urol.

[7] Soria F, Marra G, D'Andrea D, Gontero P, Shariat SF (2019). The rational and benefits of the second look transurethral resection of the bladder for T1 high grade bladder cancer. Transl Androl Urol.

[8] Sureka SK, Agarwal V, Agnihotri S, Kapoor R, Srivastava A, Mandhani A (2014). Is en-bloc transurethral resection of bladder tumor for non-muscle invasive bladder carcinoma better than conventional technique in terms of recurrence and progression?: A prospective study. Indian J Urol.

[9] Mostafid H, Babjuk M, Bochner B (2020). Transurethral resection of bladder tumour: the neglected procedure in the technology race in bladder cancer. Eur Urol.

[10] Weldon TE, Soloway MS (1975). Susceptibility of urothelium to neoplastic cellular implantation. Urology.

[11] Sylvester RJ, Oosterlinck W, Holmang S (2016). Systematic review and individual patient data meta-analysis of randomized trials comparing a single immediate instillation of chemotherapy after transurethral resection with transurethral resection alone in patients with stage pTa-pT1 urothelial carcinoma of the bladder: which patients benefit from the instillation?. Eur Urol.

[12] Brausi M, Collette L, Kurth K (2002). Variability in the recurrence rate at first follow-up cystoscopy after TUR in stage Ta T1 transitional cell carcinoma of the bladder: a combined analysis of seven EORTC studies. Eur Urol.

[13] Mancini M, Righetto M, Baggio G (2020). Spotlight on gender-specific disparities in bladder cancer. Urologia.

[14] Shariat SF, Sfakianos JP, Droller MJ, Karakiewicz PI, Meryn S, Bochner BH (2010). The effect of age and gender on bladder cancer: a critical review of the literature. BJU Int.

[15] Gupta P, Jain M, Kapoor R, Muruganandham K, Srivastava A, Mandhani A (2009). Impact of age and gender on the clinicopathological characteristics of bladder cancer. Indian J Urol.

[16] Barbosa AL, Vermeulen SH, Aben KK, Grotenhuis AJ, Vrieling A, Kiemeney LA (2018). Smoking intensity and bladder cancer aggressiveness at diagnosis. PLoS One.

[17] Megwalu II, Vlahiotis A, Radwan M, Piccirillo JF, Kibel AS (2008). Prognostic impact of comorbidity in patients with bladder cancer. Eur Urol.

[18] Croghan SM, Compton N, Manecksha RP, Cullen IM, Daly PJ (2022). En bloc transurethral resection of bladder tumors: a review of current techniques. Can Urol Assoc J.

[19] Hashem A, Mosbah A, El-Tabey NA, Laymon M, Ibrahiem EH, Elhamid MA, Elshal AM (2021). Holmium laser en-bloc resection versus conventional transurethral resection of bladder tumors for treatment of non-muscle-invasive bladder cancer: a randomized clinical trial. Eur Urol Focus.

[20] Diana P, Gallioli A, Fontana M (2022). Energy source comparison in en-bloc resection of bladder tumors: subanalysis of a single-center prospective randomized study. World J Urol.

[21] Naselli A, Introini C, Germinale F, Spina B, Puppo P (2012). En bloc transurethral resection of bladder lesions: a trick to retrieve specimens up to 4.5 cm. BJU Int.

[22] Babjuk M, Burger M, Capoun O (2022). European Association of Urology Guidelines on non-muscle-invasive bladder cancer (Ta, T1, and carcinoma in situ). Eur Urol.

[23] Truong M, Liang L, Kukreja J, O'Brien J, Jean-Gilles J, Messing E (2017). Cautery artifact understages urothelial cancer at initial transurethral resection of large bladder tumours. Can Urol Assoc J.

[24] Cheng B, Qiu X, Li H, Yang G (2017). The safety and efficacy of front-firing green-light laser endoscopic en bloc photoselective vapo-enucleation of non-muscle-invasive bladder cancer. Ther Clin Risk Manag.

[25] Hansel DE, Amin MB, Comperat E (2013). A contemporary update on pathology standards for bladder cancer: transurethral resection and radical cystectomy specimens. Eur Urol.

[26] Chen X, Liao J, Chen L (2015). En bloc transurethral resection with 2-micron continuous-wave laser for primary non-muscle-invasive bladder cancer: a randomized controlled trial. World J Urol.

[27] Liu H, Wu J, Xue S, Zhang Q, Ruan Y, Sun X, Xia S (2013). Comparison of the safety and efficacy of conventional monopolar and 2-micron laser transurethral resection in the management of multiple nonmuscle-invasive bladder cancer. J Int Med Res.

